# Cisplatin Loaded Multiwalled Carbon Nanotubes Induce Resistance in Triple Negative Breast Cancer Cells

**DOI:** 10.3390/pharmaceutics10040228

**Published:** 2018-11-13

**Authors:** Madalina Andreea Badea, Mariana Prodana, Anca Dinischiotu, Carmen Crihana, Daniela Ionita, Mihaela Balas

**Affiliations:** 1 Department of Biochemistry and Molecular Biology, Faculty of Biology, University of Bucharest, 91-95 Splaiul Independentei, R-050095 Bucharest, Romania; madalina.andreea.badea@drd.unibuc.ro (M.A.B.); anca.dinischiotu@bio.unibuc.ro (A.D.); mihaela.radu@bio.unibuc.ro (M.B.); 2 Department of General Chemistry, Faculty of Applied Chemistry and Materials Science, Politehnica University of Bucharest, 313 Splaiul Independentei, 060042 Bucharest, Romania; mariana.prodana@upb.ro; 3 CF2 Clinical Hospital Bucharest, 63 Blvd. Marasesti, 011464 Bucharest, Romania; ccrihana@hotmail.com

**Keywords:** carbon nanotubes, cisplatin, MDA-MB-231 cells, reactive oxygen species, Nrf2

## Abstract

In this paper we developed a method for multiwalled carbon nanotubes (MWCNTs) use as carriers for a drug based on platinum in breast cancer therapy. The method of functionalization involves the carboxyl functionalization of nanotubes and encapsulation of cisplatin (CDDP) into MWCNTs. The biological properties of MWCNTs loaded with CDDP (MWCNT-COOH-CDDP) and of individual components MWCNT-COOH and free CDDP were evaluated on MDA-MB-231 cells. Various concentrations of CDDP (0.316–2.52 µg/mL) and MWCNTs (0.5–4 µg/mL) were applied on cells for 24 and 48 h. Only at high doses of CDDP (1.26 and 2.52 µg/mL) and MWCNT-COOH-CDDP (2 and 4 µg/mL) cell morphological changes were observed. The cellular viability decreased only with approx. 40% after 48 h of exposure to 2.52 µg/mL CDDP and 4 µg/mL MWCNT-COOH-CDDP despite the high reactive oxygen species (ROS) production induced by MWCNTs starting with 24 h. After 48 h, ROS level dropped as a result of the antioxidant defence activation. We also found a significant decrease of caspase-3 and p53 expression after 48 h, accompanied by a down-regulation of NF-κB in cells exposed to MWCNT-COOH-CDDP system which promotes apoptosis escape and thus failing to overcome the triple negative breast cancer (TNBC) cells resistance.

## 1. Introduction

Nanomedicine is a branch of nanotechnology dedicated to nanomaterials research aimed at improving health. One of the directions in nanomedicine research is the use of different nanomaterials as drug carrier devices, in order to increase the effectiveness and safety of the drug, by reducing toxicity and side effects.

A variety of nanomaterials such as liposomes first developed in the ‘60s and organic polymers or dendrimers developed in the ‘80s have been studied extensively since their emergence and because of their high biocompatibility. Some have already been approved for clinical trials. Another type of nanomaterial that has recently generated much attention in the field of pharmaceutical research is represented by carbon nanotubes (CNTs).

Carbon nanotubes (CNTs) contributed to the development of a wide range of applications in the delivery of therapeutic agents such as peptides, proteins, nucleic acids, genes, vaccines and also in bone and neural tissue regeneration. CNTs can be functionalized with bioactive peptides, proteins, nucleic acids and drugs and used to deliver their cargos to cells and organs [1]. Because functionalized CNTs display low toxicity and are not immunogenic, such systems hold great potential in the field of nanobiotechnology and nanomedicine. In order to improve their dispersion into aqueous solutions, surface modification of CNTs, or CNTs functionalization, become a key step for their biomedical applications. The methods for CNTs modification imply non-covalent and covalent strategies. The non-covalent modification utilizes the hydrophobic nature of CNTs, especially, π–π interactions for coating with amphiphilic molecules.

These CNTs modifications offer not only water solubility but also produce functional moieties that enable linking of therapeutic agents, such as drugs and recognition molecules for biomedical applications.

Oxidation of multiwalled carbon nanotubes (MWCNTs) introduces hydroxyl (–OH) and carboxyl (–COOH) groups primarily, in the place of the nanotube structural defects and, subsequently, by a gradual degradation of MWCNTs walls [2,3].

Unmodified (pristine) MWCNTs tend to agglomerate and form so-called ‘nanotube bundles’ via weak but 3-D abundant π–π interactions [4]. Due to this phenomenon, MWCNTs are practically non-dispersible either in water or a whole set of organic solvents. Oxidative introduction of hydrophilic carboxyl and hydroxyl groups onto the MWCNTs surface significantly increases dispersibility of nanotubes in water (and other polar systems) since it allows for multiple hydrogen bonding and dipole–dipole interactions—stability of the aqueous dispersions of MWCNTs [5]. Oxidation of MWCNTs is usually performed under harsh conditions like refluxing in mixtures of HNO_3_ and H_2_SO_4_.

This process may generate multiple structural defects undesirable in many applications, especially in mechanical and electronic devices. A development of controllable, less destructive, more effective and reversible after de-bundling functionalization methods still remains a challenge [6].

Some diseases like cancer, cause a pH-decrease in certain body locations, for example, in the proximity and inside the tumour-occupied locations; a pH drop from 7.4 to 5.5 for healthy tumour cells could be observed [7].

Secondly, the needle-like shape of CNTs facilitates membrane penetration and intracellular accumulation of drugs via the “nanoneedle” mechanism that is independent of additional CNT functionalization and cell types [8].

Besides improving the water dispersibility and reducing the cytotoxicity of CNTs, surface functionalization also provides extra attachment sites for additional chemical or supramolecular loading of drugs, for targeting strategies or for imaging purposes [9,10].

Comparing with other nanocarriers such as liposomes, poly lactic-co-glycolic acid (PLGA) nanoparticles or dendrimers, studies showed that the drug delivery potential of MWCNTs is superior. Carboxylated MWCNTs show a better in vitro and ex vivo and biocompatibility profile and exhibit higher drug release capacity, especially at acidic pH corresponding to conditions existing in tumours [11].

CNTs could be promising candidates in the treatment of triple negative breast cancer (TNBC). This is a subtype of breast cancer, defined by the absence of oestrogen, progesterone and HER2 receptors. The lack of obvious targets is a major challenge in the therapy of patients with TNBC. Therapies that target these receptors cannot be used in the treatment of this type of cancer [12,13]. Chemotherapy remained the only solution for the patients with TNBC but this type of therapy is correlated with time-dependent resistance [14]. Therefore, the methods studied for treatment of TNBC target a high efficiency of drug the remove of adverse effects or resistance. There is a need to elucidate specific TNBC targets on which to rely future therapies. Our previous experiments proved that carbon nanotubes are able to penetrate the TNBC cell membrane (MDA-MB-231 cell line) and to spread into the cytoplasm [15]. In line with this, the scientific literature attests that CNTs are suitable as drug carriers by penetrating into the cancerous cells directly and keeping the drug intact unmetabolized during transport in the cell [16,17], thus addressing the major shortfalls of failed TNBC therapies.

Cisplatin (*cis*-[Pt(II)(NH(3))(2)Cl(2)] ([PtCl2(NH3)2], *cis*-Diamminedichloroplatinum (II) or CDDP), as well as carboplatin and oxaliplatin, are representative for platinum-based drugs group and are used in human cancer treatment, including breast cancer [18]. CDDP mode of cytotoxic action is mediated by adducts formation with DNA molecule, induction of oxidative stress, activation of p53 and MAPK signalling pathway [19,20]. All these mechanisms culminate in the end with the apoptotic process which is induced by tumour suppressor protein p53 or p53-related protein p73 [21].

Despite its high efficiency in early stages of treatment, CDDP has a high incidence to chemoresistance, leading in the end to therapeutic failure. When tumour cells do not respond to treatment with chemotherapeutics (CDDP), resistance mechanisms are triggered [22]. The mechanisms by which tumour cells become resistant to CDDP are intensively studied, different combinations being proposed. The most common mechanisms are based on drug inactivation by increased levels of reduced glutathione and glutathione S-transferase, reduced cellular uptake/reduced accumulation of the drug or increased drug efflux [21,22].

Combination of the CNT-based carriers and platinum drugs could offer an alternative for the treatment of TNBC if successfully pass the membrane and target the mechanism that overcome drug resistance generating cancer cell death.

In this paper, we functionalized MWCNTs with carboxylic groups followed by addition of a drug based on Pt ions (cisplatin, CDDP) and some of their biological properties in vitro on MDA-MB-231 cells were investigated in order to outline some TNBC treatment strategies.

## 2. Materials and Methods

### 2.1. Carbon Nanotubes Functionalization

MWCNTs were purchased from SigmaAldrich having more than 90% carbon basis and D × L 10–15 nm × 0.5–10 μm, produced by Catalytic Chemical Vapor Deposition (CCVD). Oxidation was achieved using a mixture of 98% sulfuric acid (Merck, Darmstadt, Germany) to obtain carboxylated multiwalled carbon nanotubes (MWCNT-COOH).

A suspension of 0.02 g MWCNT-COOH in 10.0 mL *N*,*N*-dimethylmethanamide DMF (Vetec^TM^, trademark of Sigma-Aldrich, St. Louis, MO, USA) was sonicated for 60 min. A quantity of 0.03 g of cisplatin (CDDP, Sigma-Aldrich, St. Louis, MO, USA, >99%) was dissolved in 5.0 mL of DMF and the resulting solution was added to the nanotubes suspension. The mixture was sonicated for 60 min more followed by stirring for 48 h at 50 °C. DMF was removed under vacuum at 60 °C resulting in CDDP-loaded nanotubes (MWCNT-COOH-CDDP) and CDDP excess adsorbed on the external wall. After removal of the solvent, MWCNT-COOH-CDDP were collected on a glass filter. To remove the CDDP excess from the outer surface of the CNTs, the product was washed several times with deionized water (300 mL) until all adsorbed CDDP were removed according to Pt analysis on the filtrate aliquots by ICP-MS method. The collected MWCNT-COOH-CDDP were dried in an oven at 80 °C [23].

### 2.2. Quantification of Platinum Ions

Platinum concentration was quantified using inductively coupled plasma mass spectrometry (ICP-MS) that is a versatile method for sub-part per million quantifications of plentiful elements like platinum, calcium, rhodium and palladium.

The amount of platinum ions released in simulated body fluid (SBF) was highlighted by inductively coupled plasma mass spectrometry (ICP-MS). The mass spectrometer was provided by Perkin Elmer Company (Shelton, DC, USA), an ELAN DRCe model. The detection limit was 0.001 μg·g^−1^.

It is a type of emission spectroscopy technique employing inductively coupled plasma to produce excited atoms or ions of metals, each exhibiting a characteristic wavelength upon emission of electromagnetic radiations [24]. The energy transfer from electrons when they fall to ground state is unique to each element. The compounds under investigation are digested using numerous techniques like treatment with single or combinations of acids and heating process for extraction of desired metals. Also, the interference of other extraneous components in this method is found to be negligible [25,26]. Hence, a simple and rapid ICP-MS method was developed and validated for determination of CDDP levels in biological samples.

### 2.3. FTIR Spectroscopy

The structure of the samples after functionalization with CDDP was identified with FTIR spectroscopy using an ATR system Spectrum 100 equipment in 500–4500 cm^−1^ range with 4 cm^−1^ resolution and 32 scans from Perkin Elmer Company.

### 2.4. Scanning Electronic Microscopy

The samples were characterized by scanning electronic microscope (SEM) model Quanta 650 FEG from FEI company (Hillsboro, OR, USA) equipped with an EDS module (energy dispersive spectra). Electron acceleration voltage was 10 kV. Elemental mapping analysis was performed, to put in evidence the existence of elements present in each sample by energy-dispersive X-ray spectroscopy (EDX). EDX data was recorded at 10 kV using the SEM electron beam and the X-ray spectrometer mounted in the SEM equipped with a silicon drift detector (SDD) at a resolution of 139 eV in accordance with ISO 15632:2002 standard.

### 2.5. Cell Culture

MDA-MB-231 cell line (ATCC HTB-26, Manassas, VA, USA), a human breast adenocarcinoma cell line representative for TNBC, was maintained in culture at 37 °C in humidified atmosphere with 5% CO_2_. The cells were cultured in 75 cm^2^ culture flasks using Dulbecco’s Modified Eagle Medium (DMEM, cat. no. 31600-083, Gibco by Thermo Fisher Scientific, Carlsbad, CA, USA) supplemented with 3.5 g/L glucose, 1.5 g/L NaHCO_3_, 1% antibiotics and antimycotics solution (cat. no. A5955, Sigma-Aldrich, St. Louis, MO, USA) and 10% foetal bovine serum (cat. no. 10270-106, origin South America, Gibco by Thermo Fisher Scientific, Carlsbad, CA, USA). Similar, normal MRC-5 cells (human lung normal fibroblasts) (ATCC CCL-171, Manassas, VA, USA) were cultivated in Eagle’s Minimum Essential Medium (MEM, cat no. 41500-018, Gibco, Thermo Fisher Scientific, Carlsbad, CA, USA) supplemented with 1.5 g/L NaHCO_3_, sodium pyruvate 100 mM (cat. no. 11360-039, Gibco, Thermo Fisher Scientific, Carlsbad, CA, USA), 1% antibiotics and antimycotics solution (cat. no. A5955, Sigma-Aldrich, St. Louis, MO, USA) and 10% foetal bovine serum (cat. no. 10270-106, origin South America, Gibco by Thermo Fisher Scientific, Carlsbad, CA, USA).

### 2.6. Cell Treatment

Breast cancer and non-tumour cells were exposed to various concentrations of CDDP, MWCNT-COOH and MWCNT- MWCNT-COOH-CDDP for 24 and 48 h. The tested concentrations were 0.5, 1, 2 and 4 µg/mL for MWCNTs and respectively 0.316, 0.63, 1.26 and 2.52 µg/mL for CDDP. Untreated cells were used as control.

### 2.7. Cell Viability

The cellular viability, after CDDP and MWCNT exposure, was analysed by MTT test. Briefly, MDA-MB-231 cells were seeded in 24-well plates at a density of 4 × 10^4^ cells/mL and after their adherence, they were exposed to treatment as described above. After 24 and 48 h, culture medium was removed and the cells were treated with 1 mg/mL 3-(4,5-dimethylthiazol-2-yl)-2,5-diphenyltetrazolium bromide (MTT) solution (M2128, Sigma-Aldrich, St. Louis, MO, USA). Formazan crystals formed after 2 h incubation at 37 °C were solubilized in isopropanol. In the end, the optical density was read at 595 nm.

### 2.8. The Level of Lactate Dehydrogenase (LDH) Released in Culture Medium

Plasma membrane damage and nanoparticles cytotoxicity, were evaluated by measuring LDH releasing in culture medium. In normal conditions, LDH has a cytoplasmic localization but when the integrity of plasma membrane is altered, LDH enzyme pass in culture medium. The LDH activity was measured at 24 and 48 h after MDA-MB-231 and MRC-5 cells treatment using the “Cytotoxicity Detection Kit (LDH)” (cat. no. 14115700, Roche, Mannheim, Germany) according to manufacturer’ instructions (ver. 10). Briefly, a volume of 50 µL of cell culture medium was incubated with 50 µL reaction mixture for 15 min at room temperature and the absorbance of samples was measured at 490 nm using a Flex Station 3 Microplate Reader (Molecular Devices, San Jose, CA, USA).

### 2.9. Cell Morphology Examination and Fluorescence Labelling of Actin Cytoskeleton

The MDA-MB-231 and MRC-5 cell morphology was examined after the exposure to various concentrations of MWCNTs (0.5–4 µg/mL) and CDDP (0.316–2.52 µg/mL) by optical and fluorescence microscopy using an Olympus IX73 (Olypmus, Tokyo, Japan) inverted microscope equipped with a Hamamatsu camera (A3472-06, Hamamatsu, Japan). The images were acquired using the cellSens Dimension software (ver 1.11, Olypmus, Tokyo, Japan). Cytoskeleton actin integrity was evaluated after the treatment of MDA-MB-231 cells with two concentrations of MWCNTs and CDDP: 1 µg/mL/0.63 µg/mL CDDP and 2 µg/mL/1.26 CDDP. Actin filaments were labelled with Alexa Fluor 488 phalloidin dye (A12379, Molecular Probes by Life Technologies, Carlsbad, CA, USA). After the medium was removed, the cells were washed with phosphate saline buffer (PBS) and then fixed with 4% paraformaldehyde for 10 min at 4 °C. Further, cell membrane was permeabilized through incubation with a 0.1% TRITON X-100 in 2% albumin serum bovine (BSA) solution, for 45 min. Actin filaments were labelled with 150 nM Alexa Fluor 488 solution at room temperature for 45 min. A concentration of 2 µg/mL Hoechest dye solution was used to stain cell’s nuclei (10 min, room temperature). Finally, the labelled cells were examined under a fluorescence microscope.

### 2.10. Reactive Oxygen Species (ROS) Production

To quantify the ROS production in tumour and non-tumour cells, 10^4^ cells/well were seeded in 96-black well plates (165305, Thermo Scientific Nunc, Rochester, NY, USA) and incubated overnight. After 24 h, respectively 48 h, of exposure to various concentrations of treatment, the culture medium was removed and the cells were incubated for 30 min with 50 µM 2′,7′-dichlorofluorescindiacetate (H2DCF-DA, Sigma-Aldrich, St. Louis, MO, USA) at 37 °C. Upon interaction with ROS, the H2DCF-DA is converted to 2′,7′-dichlorofluorescein (DCF), a fluorescent compound which was detected through fluorescence spectroscopy at ex. 485 nm, em. 520 nm.

### 2.11. Preparation of Cellular Lysate

The breast adenocarcinoma and normal MRC-5 cells were collected from culture dishes after treatment and centrifuged for 5 min at 1500 rpm. Cell sediment was washed and then resuspended in PBS. Cells lysis was obtained by sonication on ice, three times for 30 s using a UP50H ultrasonicator (Hielscher Ultrasound Technology, Teltow, Germany) at 80% amplitude, 1 cycle. After the centrifugation (10 min, 3000 rpm, 4 °C), the supernatant was collected and used for further determinations. The protein concentration was measured by Bradford method using BSA as standard protein [27].

### 2.12. Reduced Glutathione (GSH) Content

GSH concentration was assessed after the exposure of breast cancer cells to 1, 2 µg/mL MWCNTs and 0.63, 1.26 µg/mL CDDP for 24 h and 48 h using Glutathione Assay Kit (CS0260, Sigma-Aldrich, St. Louis, MO, USA). Cellular lysate was first deproteinized with an equal volume of 5% 5-sulfosalicylic acid (S2130-500G, Sigma-Aldrich, St. Louis, MO, USA). The supernatant resulted after samples’ centrifugation (10 min, 10,000 rpm, 4 °C) was transferred in a 96-well plate, treated with a solution of 5,5′-dithiobis (2-nitrobenzoic acid) (DTNB; D8130, Sigma-Aldrich, St. Louis, MO, USA) in assay buffer and incubated for 10 min at room temperature. 5-thio-2-nitrobenozoic acid (TNB) formed after GSH oxidation was detected spectrophotometrically at 405 nm. A solution of 200 µM reduced glutathione (G6529-25G, Sigma-Aldrich, St. Louis, MO, USA) was used as standard for calibration curve.

### 2.13. Glutathione S-Transferase (GST) Activity

GST activity was assessed after MDA-MB-231 cells exposure to MWCNTs and CDDP for 24 h, respectively, 48 h. A volume of 50 µL sample was mixed with 20 µL 1-Chloro-2,4-dinitrobenzene (CDNB) 25 mM, 100 µL GSH 20 mM, 200 µL phosphate buffer 0.1 M, pH 7.1 and distilled water up to 1 mL. The increase of absorbance at 340 nm, directly proportional with GST activity of the sample, was followed within 5 min through conjugation of GSH with CDNB [28]. The specific activity of GST was estimated in U/mg protein. One unit of GST activity was defined as the amount of enzyme that catalysed the transformation of 1 μmol of CDNB in conjugated product per min. The CDNB concentration was calculated using the molar extinction coefficient (ε_CDNB_ = 9.6 ×10^3^ M^−1^·cm^−1^) and results were expressed as percentage of control.

### 2.14. Nuclear Factor E2-Related Factor 2 (Nrf2) Protein Expression

The expression of Nrf2 (Nuclear factor (erythroid-derived 2)-like 2), caspase-3, tumour protein p53, Beclin-1 and NF-κB (nuclear factor kappa-light-chain-enhancer of activated B cells) proteins was evaluated after cells exposure to CDDP, MWCNT-COOH and MWCNT-COOH-CDDP by Western blot technique. Thus, 25 µg of protein from treated and untreated samples were loaded on a 10% SDS-polyacrylamide gel and separated for 2 h at 90 V in TRIS-glycine buffer. Further, the proteins were transferred from the gel to an Immuno-Blot PVDF membrane (cat. no. IPVH00010, Merck, Darmstadt, Germany). The transfer step was performed in a wet system (TRIS-glycine-methanol buffer), for 90 min. The membranes were developed using WesternBreeze Chromogenic Anti-Rabbit and Anti-Mouse Kits (WB7105, WB7103, Invitrogen, Carlsbad, CA, USA), polyclonal anti-Nrf2 (Santa Cruz, Biotechnology, Dallas, TX, USA, sc-722), anti-caspase-3 (Santa Cruz, Biotechnology, Dallas, TX, USA, sc-7148), anti-p53 (Santa Cruz, Biotechnology, Dallas, TX, USA, sc-6243), anti-beclin-1 (Santa Cruz, Biotechnology, Dallas, TX, USA, sc-48381), anti-NF-κB (Santa Cruz, Biotechnology, Dallas, TX, USA, sc-109) specific antibodies and monoclonal anti-β-actin antibody (A1978, Sigma-Aldrich, St. Louis, MO, USA), following the manufacturer’s instructions. Protein bands were revealed using BCIP/NBT substrate and were visualized with the ChemiDoc Imaging System (Bio-Rad, Hercules, CA, USA). Bands densitometry was realized with Image Lab software (ver. 5.1, Bio-Rad, Hercules, CA, USA) and β-actin was used as reference protein.

### 2.15. Statistical Analysis

All experiments were done in triplicate. The biological data are calculated as an average of three replicates ± standard deviation and represented in percent. All sample values were related to control (untreated cells) value which was considered 100% and statistically compared using Student’s *t*-test. The differences between control and samples values were considered significant at *p* < 0.05, highly significant at *p* < 0.01 and extremely significant at *p* < 0.001.

## 3. Results and Discussion

### 3.1. FTIR Measurements

**Figure 1:**
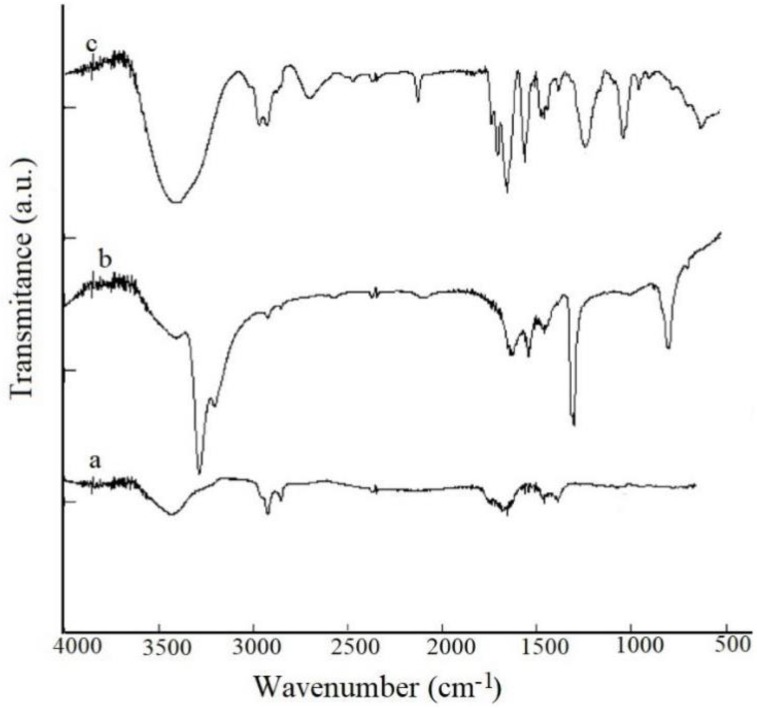
FTIR spectra for (**a**) MWCNT-COOH; (**b**) CDDP; (**c**) MWCNT-COOH-CDDP.

FTIR spectroscopy (Figure 1) put in evidence the presence of functional groups on the surface of the carboxylated nanotubes. Thus, the peaks observed at 1150 and 1738 cm^−1^ are related to C–O and C=O [29]. Observed peaks which appeared in region of 2858 cm^−1^ and 2930 cm^−1^ are due to C–H stretching bonds and the peak that appear at 3450 cm^−1^ assigned to O–H (carboxylic acid) group.

The covalent binding of CDDP to MWCNT-COOH is revealed by the peaks that appear between 500 and 1600 cm^−1^. Demonstration of drug encapsulation in MWCNTs is evidenced by the presence of specific bands Pt–N at 724 cm^−1^ and a new band at approximately 600 cm^−1^ that can be assigned to P–O [30].

### 3.2. SEM Characterization

**Figure 2:**
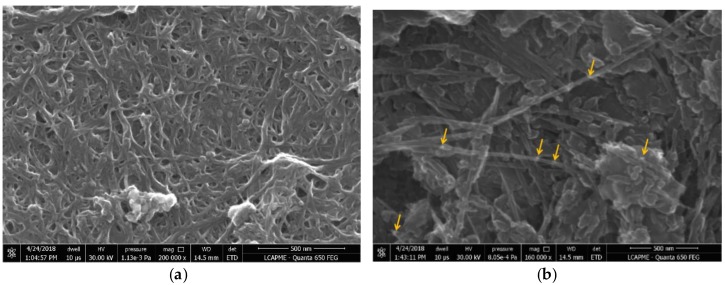
SEM morphologies for: (**a**) MWCNT-COOH and (**b**) MWCNT-COOH-CDDP. Yellow arrows indicate some examples of granular structures of MWCNT-COOH-CDDP.

SEM images for MWNT-COOH and MWCNT-COOH-CDDP are given in Figure 2a,b, respectively. The samples were characterized using a scanning electron microscope from FEI, model Quanta FEG650. The morphologies of the samples are shown in Figure 2. Figure 2a shows the smooth surface of the MWCNT-COOH which presents some agglomerations. Figure 2b represents the morphology of the sample that was functionalized with drug, having a granular structure in a more dispersed form. The platinum appears like lighter spots in the images because of the higher atomic number (Z) compared with carbon.

**Figure 3:**
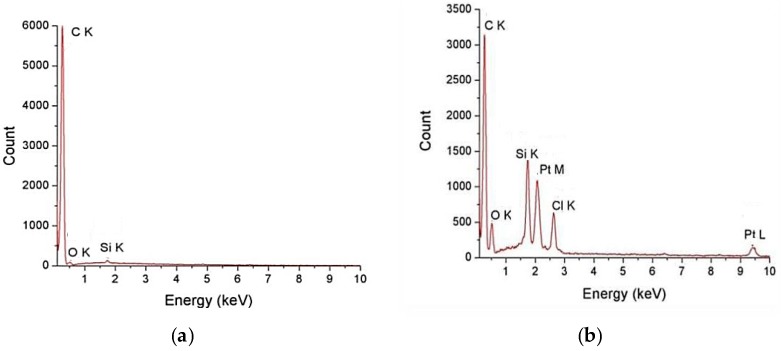
EDX spectra for: (**a**) MWCNT-COOH and (**b**) MWCNT-COOH-CDDP.

Figure 3 represents the EDX spectra for samples. The spectra of MWCNT-COOH presents peaks that are characteristic for C, O, Si. In the spectra of MWCNT-COOH-CDDP sample, C, O, Pt and Si peaks appears. Pt peak is due to the CDDP functionalization.

### 3.3. ICP-MS Analysis

The ICP-MS measurements indicate the concentration of Pt ions in the domain of µg/mL.

For ICP-MS analysis, all samples (typically 1 mL) were digested in 100 mL concentrated nitric acid ULTRAPURE (Merck, Darmstadt, Germany). Acid digestion was done in a well determined volume of HNO_3_ 65%. After digestion, the samples were diluted 100 times and liquid fractions were analysed. Platinum (20 mg/L) as internal standard was applied to analyse solutions in ICP-MS. The amount of drug (CDDP) encapsulated in MWCNT-COOH sample is 191.2 (µg/mL).

This low release rate is probably due to the fact that the drugs covalently bonded to the surface of MWCNTs and breaking the barrier of C-N and C-C bonds it will take some time. Not all the bonds will be favourable breaking as we know that covalent bond is a very stable one. Our experiments evidenced that the loading percent for cisplatin on MWCNT-COOH was 38.2%, higher than the loading obtained for SWCNT-COOH.

### 3.4. In Vitro Release of CDDP from MWCNT-COOH-CDDP Complex

This evaluation of CDDP concentration was done using a diffusion technique in a dialysis bag.

Before dialysis was performed the bag used was kept 12 h in deionized water to ensure the wetting of the membrane. To obtain the release curve of CDDP from MWCNT-COOH-CDDP sample, this complex (containing 0.5 mg of CDDP) was dispersed in deionized water (200 µL). These studies were performed in PBS. The dialysis take place at 12,000~14,000 Da (which allowed the free exchange of CDDP and release media) and immersed into PBS (pH 7.4-the pH of the human body) as release medium (400 mL). A specific volume (2 mL) of sample was collected from the release medium at regular intervals (3 h, 6 h, 9 h, 12 h, 15 h, 18 h, 24 h, 48 h, 72 h, 96 h) and their platinum contents were determined using ICP-MS to evaluate the CDDP released from MWCNT-COOH-CDDP. Also, the experiment was carried out in PBS at a pH 5.5 (typical cancerous environment pH).

### 3.5. In Vitro Release of CDDP from MWCNT-COOH-CDDP in Neutral and Weakly Acidic Conditions

**Figure 4:**
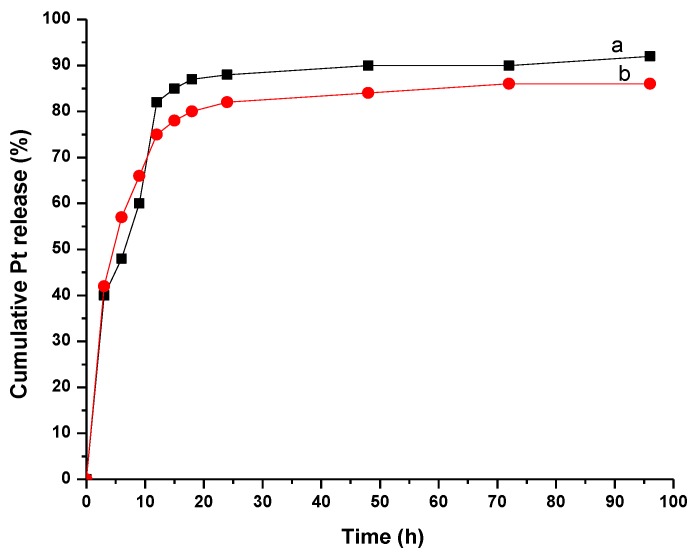
Pt release for MWCNT-COOH-CDDP in PBS at (**a**) 5.5 and (**b**) 7.4 pH values.

Cancer needs an acidic and poor oxygen environment to expand [31]. The profile of curve shows the release of CDDP from the sample at two pH values: 5.5 and 7.4 and it was observed that the values are comparable. Rapid release of CDDP from MWCNT-COOH-CDDP took place mainly in the first hours (Figure 4), MWCNTs having the diameter between 10–15 nm. Approximately 75% and 83% of encapsulated CDDP were released after 10 h of immersion in PBS at pH 7.4 and pH 5.5 respectively. This was in accordance with the literature data which reported that drug release mainly occurs after 48 h of immersion in PBS [32]. There is no significant interaction between CDDP and the walls of MWCNTs because the release of the drug was almost complete. For single walled carbon nanotubes [33] it took almost 20 h to release half amount of CDDP because of their small diameter (2–5 nm). The release rate of CDDP encapsulated into nanotubes can be controlled by chemical modification of the functional groups attached to the tubes [34]. As the CNTs were immersed in an aqueous medium, water could enter and exit freely the tubes. CDDP is soluble in water compared with MWCNTs, so it could be displaced from nanotubes because of the preference for water in opposite of being encapsulated inside hydrophobic MWCNTs. In order to modify the rate of release it is interesting to find new ways of modifying the open ends of carbon nanotubes for slowing down the CDDP release.

### 3.6. Cytotoxicity of Drug Loaded MWCNT

**Figure 5:**
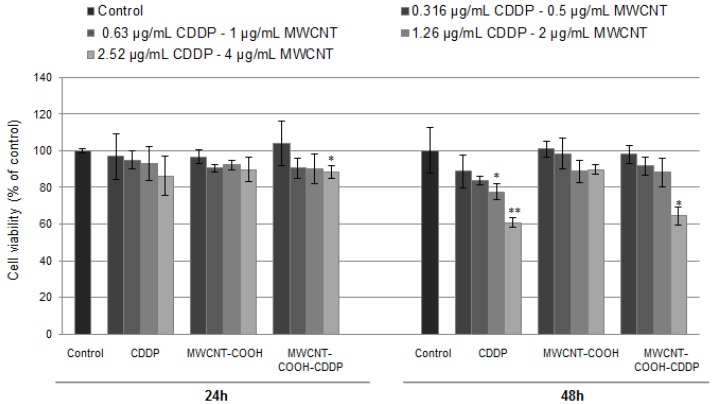
MDA-MB-231 breast cancer cell viability after 24 and 48 h of exposure to various concentrations of MWCNTs (0.5–4 µg/mL) and CDDP (0.316–2.52 µg/mL). The results are calculated as the mean ± SD of 3 replicates and represented relative to control (untreated cells—100% cellular viability). * *p* < 0.05, ** *p* < 0.01 vs. control.

Cellular viability of breast cancer cells was evaluated after the exposure to 0.5–4 µg/mL MWCNTs and 0.316–2.52 µg/mL CDDP for 24 h and 48 h. Based on reduction by mitochondrial dehydrogenases, MTT test is an indicator of the cellular viability and cell metabolic activity. The results indicate that cellular viability presented no modifications after 24 h of exposure except for the highest tested concentration of MWCNT-COOH-CDDP (Figure 5). After 48 h, cellular viability significantly decreased up to 60%, respectively 64%, relative to control in the samples treated with 2.52 µg/mL CDDP, respectively 4 µg/mL MWCNT-COOH-CDDP, in contrast to MWCNT-COOH sample which had no significant effects on breast cancer cell viability showing no toxicity.

The effects induced by CDDP on cancer cells viability are intensively studied. In the scientific literature, the anticancer capacity of CDDP is evaluated through the half-maximal inhibitory concentration (IC_50_), the concentration at which cellular viability decreases to 50%. We found that IC_50_ value of CDDP slightly vary between studies. Wang et al. [35] treat the MDA-MB-231 cells with CDDP and found an IC_50_ value of 25.28 µM (meaning 7.5 μg/mL). Also, the study showed that a dose of 10 µM (3 µg/mL) CDDP decreased breast cancer cell viability with approx., 25–30% after 48 h. Using the same cell line, Pauzi et al. [36] reported in their paper a decrease with about 40% of cell viability after 48 h for a dose of 3 µg/mL CDDP and an IC_50_ value of 23.0 µM (6.9 µg/mL). In accordance with the last study, we obtained a similar decrease of breast cancer cell viability after 48 h with approx. 40% for a dose of 2.5 µg/mL. In combination with MWCNTs the level of cell viability was quite similar, only with 4% higher. So, we found that loading of CDDP in MWCNTs does not change the potency of drug in altering the breast cancer cell metabolic activity.

**Figure 6:**
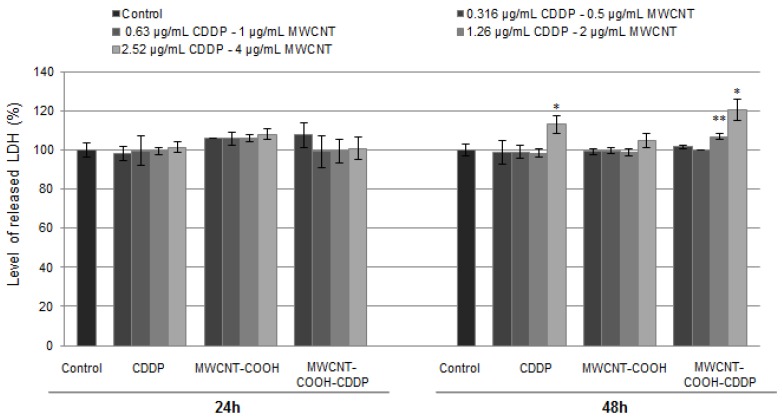
The level of LDH released in culture medium by breast cancer cells after 24 h and 48 h of exposure to various concentrations of MWCNTs (0.5–4 µg/mL) and CDDP (0.316–2.52 µg/mL). The results are calculated as the mean ± SD of 3 replicates and represented relative to control (untreated cells). * *p* < 0.05, ** *p* < 0.01 vs. control.

The results revealed by MTT test were in accordance with ones resulted from the LDH assay, which was performed in the same conditions as cellular viability test. LDH is a cytoplasmic enzyme which is released in extracellular medium when the membrane is damage. The LDH leakage is also considered an indicator of necrosis. Similar to MTT test, the level of LDH has remained unchanged after 24 h of exposure, suggesting no modifications of membrane integrity. After 48 h, high doses of MWCNT-COOH-CDDP (2 µg/mL and 4 µg/mL) and CDDP (2.52 µg/mL) induced a significant increase of LDH activity in culture medium (Figure 6). We noticed here that, the level of LDH release was higher with 7% when the cells were treated with 2 and 4 µg/mL MWCNT-COOH-CDDP, relative to the corresponding concentration of CDDP, indicating a more pronounced cytotoxic effect of the combination MWCNT-CDDP.

The cytotoxic action of CNTs loaded with drugs through the release of LDH in culture medium was demonstrated also by Zheng et al. [37]. Thus, after the exposure of K562 cells to single-walled carbon nanotubes loaded with SNX-2112 (an inhibitor of Hsp90 protein) and the individual components, the level of LDH was higher for the drug-loaded nanotubes than the free drug, indicating a superior efficiency of the drug when loaded into CNTs.

Thus, we put in evidence that in combination with MWCNTs, the CDDP induced toxicity in MDA-MB-231 cells even at lower concentrations (1.26 µg/mL).

Similar to MDA-MB-231 cells, the exposure of normal cells (MRC-5 cells) to 0.316–2.52 µg/mL CDDP and 1–4 µg/mL MWCNTs induced no modifications on LDH level released in culture medium after 24 h. Instead, starting with 48 h, the level of LDH was significantly increased when the normal cells were exposed to the highest dose of CDDP (2.52 µg/mL). Interesting, the level of LDH enzyme remained near to control at the correspondent concentration of MWCNTs (4 µg/mL) indicating a less cytotoxic tendency of MWCNT-COOH-CDDP sample (Figure S1).

### 3.7. Cell Morphology and Actin Cytoskeleton Integrity

**Figure 7:**
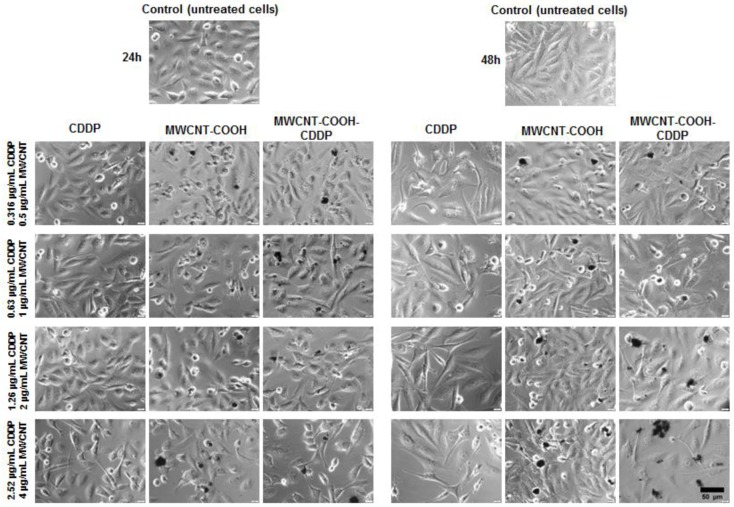
Bright-field images presenting the MDA-MB-231 cells morphology after exposure to various concentrations of MWCNTs (0.5–4 µg/mL) and CDDP (0.136–2.52 µg/mL) for 24 h and 48 h. Control represents untreated cells. Aggregates of CNTs are visible over the cell fields (black dots). Scale bar: 50 µm.

Cell morphology and actin cytoskeleton integrity were analysed after 24 h and 48 h of exposure to various concentrations of MWCNTs (0.5, 1, 2, 4 µg/mL) and CDDP respectively (0.316, 0.63, 1.26, 2.52 µg/mL). The optical microscopy images showed slight morphological changes of cells at high concentrations of MWCNTs (2, respectively, 4 µg/mL) and CDDP (1.26, respectively 2.52 µg/mL). In culture medium, MWCNTs had the tendency to form aggregates with various dimensions and to attach to cellular membrane (Figure 7). In addition, more pronounced morphological alterations were noticed after 48 h of exposure comparative to 24 h interval. In comparison with the normal epithelial-like morphology, MDA-MB-231 cell became more elongated after treatment and their density was visible reduced.

Exposure of normal cells to the same concentrations of CDDP (0.316–2.52 µg/mL) and MWCNTs (1–4 µg/mL) revealed important modifications in cell morphology and density after the treatment with CDDP and MWCNT-COOH-CDDP even at 24 h. Moreover, the effects were more pronounced after 48 h of exposure. In comparison with MWCNT-COOH-CDDP system, an important reduction of cell density accompanied by a gathered morphology was observed when the cells were exposed to 1.26 and 2.52 µg/mL CDDP (Figure S2). Slighter morphological modifications were revealed after the incubation of cells with MWCNT-COOH-CDDP sample. The significantly modifications revealed by optical microscopy were in correlation with the higher level of LDH released in culture medium after MRC-5 cells exposure to 2.52 µg/mL CDDP.

**Figure 8:**
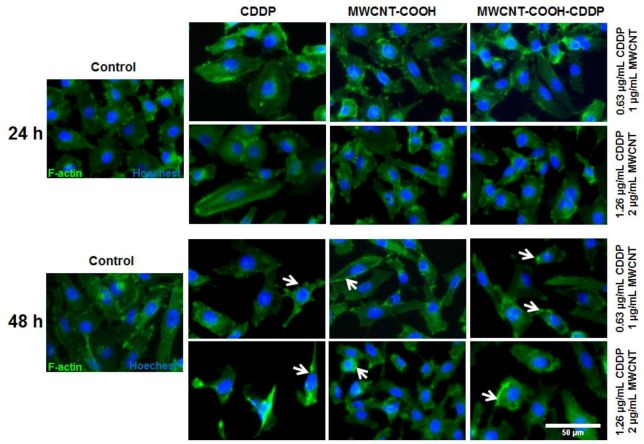
Immunofluorescence labelling of MDA-MB-231 cells after 24 h and 48 h of exposure to 1, 2 µg/mL MWCNTs and 0.63, 1.26 µg/mL CDDP. Green fluorescence (Alexa Fluor 488 phalloidin dye) indicates F-actin filaments and blue labelling (Hoechest) corresponds with cells nuclei. White arrows indicate filaments actin modifications. Scale bar: 50 µm.

Two concentrations of CDDP of 0.63 and 1.26 µg/mL have been chosen for the further experiments. Several morphological changes were highlighted by the actin cytoskeleton dynamic after 48 h of exposure. Thus, exposure of MDA-MB-231 cells to 1, 2 µg/mL MWCNT-COOH-CDDP and 0.63, 1.26 µg/mL CDDP induced F-actin filaments aggregation and loss of stress fibres (Figure 8).

Similar with our study, changes in cell morphology and disruption of actin filaments after treatment with CDDP were also observed in other studies. Zeidan et al. (2008) [38] investigated the effects induced by CDDP on breast cancer cells (MCF-7) and noticed significant changes in actin organization. Cytoskeleton remodelling was suggested by the appearance of cortical stress fibres, destabilization of membrane anchoring of actin filaments and inactivation of ezrin, a protein implicated in regulation of cytoskeleton dynamics by cross-linking actin filaments to the plasma membrane. Baribeau et al. (2014) [39] also reported a clear morphology change from an epithelial morphology to a more elongated morphology after exposure of ovarian cancer cells to CDDP.

Mechanistically, some studies revealed that the elevation of ROS levels might be responsible for changes in the actin cytoskeleton structure [40].

### 3.8. Oxidative Stress

ROS represent reactive molecules and free radicals derived from oxygen which are permanently produced in living cells and neutralized by antioxidant defence mechanisms. However, under certain conditions, the rate of ROS production goes higher, antioxidants cannot eliminate these molecules and, as a consequence, oxidative stress installs [41,42].

**Figure 9:**
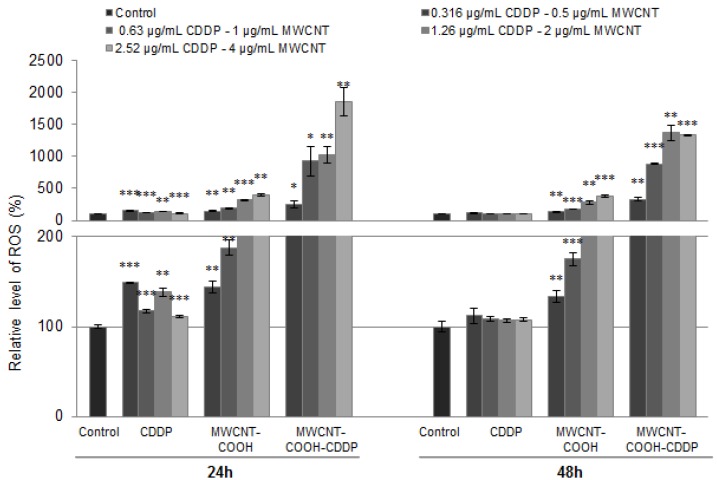
Relative level of ROS production after the exposure of MDA-MB-231 cells to MWCNTs (0.5–4 µg/mL) and CDDP (0.316–2.52 µg/mL) for 24 h and 48 h. The results are calculated as the mean ± SD of 3 replicates and represented relative to control. * *p* < 0.05, ** *p* < 0.01, *** *p* < 0.001 vs. control. The lower graph presents a magnified image of the scale range between 0–200 from the upper graph.

To evaluate the oxidative stress, specific markers such as intracellular ROS production, GSH content, GST activity and Nrf2 protein expression were analysed after 24 h and 48 h of treatment. The ROS production, after exposure to 0.5, 1, 2, 4 µg/mL MWCNTs and 0.316, 0.63, 1.26, 2.52 µg/mL CDDP, increased in a dose-dependent manner. As shown in Figure 9, the highest level of ROS was generated in breast cancer cells exposed to MWCNT-COOH-CDDP at 24 h in comparison with MWCNT-COOH and free drug. After 48 h, the generation of ROS was slightly inhibited and decreased comparative to 24 h suggesting an antioxidant response of breast cancer cells to oxidative stress. Similar results were obtained in one of our previous studies [15]. There, we have shown that treatment of MDA-MB-231 cells with MWCNT-COOH, carboplatin and MWCNT-COOH loaded with carboplatin, induced an increase of superoxide anion level which was higher for MWCNT-COOH loaded with carboplatin, in comparison with the corresponding concentration of free drug.

Although it is already known that cancer cells are characterized by an elevated level of ROS [43], generation of ROS after CDDP treatment could represent an important parameter to evaluate drug efficiency.

We found that the lowest level of ROS was produced in cells treated with free drug. Previous research studies postulated that the primary cytotoxic mechanisms of CDDP is DNA damage and the subsequent induction of apoptosis. However, other studies demonstrated that CDDP induced a mitochondrial-ROS response that contributed to its cytotoxicity [44]. Exposure of CDDP to HK-2 cells revealed that intracellular generated ROS are located in mitochondria (mitochondrial ROS) in a percentage of 70% of the total ROS. As a result, mitochondria and mitochondrial DNA are damaged. Morphological changes in mitochondria were correlated with fragmentation, mitochondrial membrane potential disruption and down-regulation of mitochondrial stability markers [45]. The ROS generated during drug metabolism after exposure to platinum complexes also led to oxidative modifications of unsaturated fatty acids, inducing peroxidation with increased levels of hydrogen peroxide and a prostaglandin derivative, 8-isoprostane. Moreover, it was shown that the ER-positive cells (MCF7) are more susceptible to platinum drugs action than the ER-negative cells (MDA-MB-231) [46]. Another mechanism through CDDP can induce ROS production was described by Itoh et al. (2011) [47] and it is underlined by NADPH oxidase activation.

On the other hand, CNTs directly induce ROS formation into the cells. Several factors are involved in ROS generation by CNTs, including surface modifications, surface area, surface reactivity, shape, length, agglomeration and number of layers of CNTs [48,49]. Usually, commercial CNTs, either SWCNTs or MWCNTs, have a high number of transition elements that cause dose-dependent increases in ROS formation. The metals have the potential to convert hydrogen peroxide and superoxide anions to hydroxyl free radicals causing oxidative stress [50]. We showed that MWCNT-COOH induced a higher ROS production in MDA-MB-231 cells compared to free CDDP suggesting thus that CNTs might have exacerbated induction of intracellular ROS generation possible due to surface reactivity. In combination with CDDP, MWCNT-COOH have generated even more ROS, probably due to a synergic action of the two components which also might indicate the efficiency of MWCNTs functionalized with carboxyl groups in delivery of CDDP into TNBC cells.

However, the high amount of ROS did not induce a decrease of cell viability in MDA-MB-231 cells but caused significant actin cytoskeleton alteration and changes of some protein expression as we further show.

Similar outcomes with our study were obtained by Ju et al. [51]. This group demonstrated that MWCNTs failed to induce apoptosis, cell cycle arrest or DNA damage in A549 cells at doses of 0.3, 3 and 30 µg/mL at 24 and 48 h. Only, in the case of treatment with 300 µg/mL MWCNTs for 24 h, the percentage of early apoptotic cells increased from 3% to 7% and after 48 h, a 2-fold increase in the percentage of both early apoptotic and apoptotic cells was observed. They also detected elevated ROS levels in association with changes in the actin structure and altered expression of 106 proteins [51]. In the same time, human skin fibroblast cells turned out to be more sensitive. After 48 h exposure to the same MWCNTs, at a dose of only 0.06 µg/mL, proliferation was reduced by 50% and apoptosis/necrosis and G2/M block increased by 2-fold [52].

Previous studies have also demonstrated that tumour cells are more resistant to ROS attack compared with normal cells owning a strong ROS scavenging system to maintain their homeostasis and immortal [53]. After 24 h the ROS level significantly increased compared with control in both normal and cancerous cells and the increase was more pronounced for normal cells by about 28-fold compared with 15-fold in cancer cells for the highest tested dose (Figure S3). Mostly, the increase of ROS was dose-dependent. After 48 h a slight diminish of ROS level was observed only for the two highest doses.

According to literature, cancer cells can adapt to high ROS circumstance and sometimes, ROS induce cancer cell proliferation which might explain the high cell viability level observed in our experiment. This characteristic of cancer cells allows to acquire a resistance to oxidative stress conditions compared to normal cells. The signalling pathways activated by elevated levels of ROS in cancer cells cause not only up-regulation of several genes involved in cellular proliferation and evasion of apoptosis but also an increase metabolic rate in cancer cells [54]. In tissues, the increase of intracellular ROS levels resulted from tumorigenic events such as oncogene activation, metabolic alterations or macrophage infiltration can promote tumour formation or progression [43].

LDH is a marker for necrosis and is expressed in cell medium when cellular membrane is damaged. Thus, we explain the insignificant LDH release and unaltered morphology as additional proofs of oxidative stress counteraction and resistance of cancer cells. By comparison in normal cells, no significant change of LDH level was observed except for the highest dose of free CDDP (increased by ~16%).

On the other hand, excessive increase in intracellular ROS production as mediated by drugs, could cause cell cycle arrest, senescence or cancer cell death but may be also counteracted by the tumour cells through an increase in the expression of endogenous antioxidants [43].

**Figure 10:**
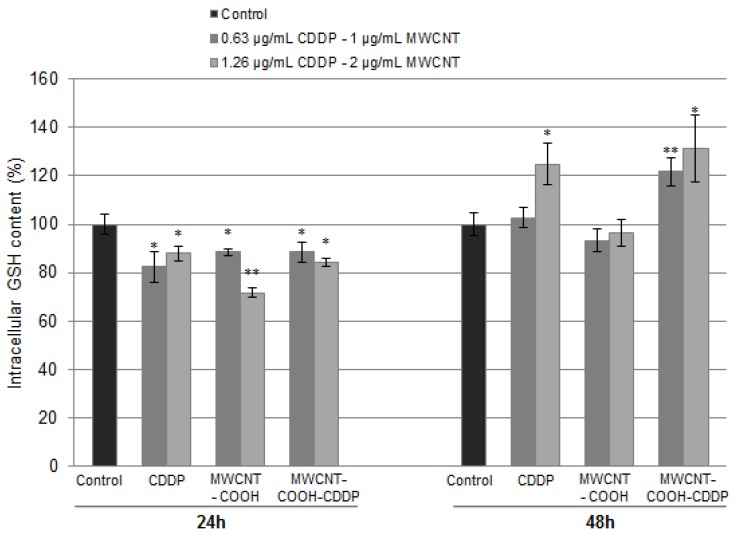
Intracellular GSH content after breast cancer cell exposure to MWCNTs (1, 2 µg/mL) and CDDP (0.63, 1.26 µg/mL) for 24 h and 48 h. The results are calculated as the mean ± SD of 3 replicates and represented relative to control. * *p* < 0.05, ** *p* < 0.01 vs. control.

We further evaluated, the content of GSH, the main cellular antioxidant, after exposure of cells to 1, 2 µg/mL MWCNTs and 0.63, 1.26 µg/mL CDDP. After 24 h, a significantly decrease of GSH concentration was observed for all the tested samples more pronounced for MWCNT-COOH (decrease by 29%). The decreased GSH concentration and the high level of ROS represent an evidence of oxidative stress occurrence in breast cancer cells. Elevated levels of GSH were observed when the cells were exposed to treatment for 48 h suggesting an activation of defence mechanisms to counteract the ROS production. The level of GSH increased with 24% and 31% when the cells were exposed for 48 h to 1.26 µg/mL CDDP, respectively, 2 µg/mL MWCNT-COOH-CDDP (Figure 10) related to control cells. By comparison, the level of GSH in cells treated with free drug was lower or unchanged in ones treated with unloaded MWCNTs indicating a higher antioxidant defence for CDDP-loaded MWCNT-COOH.

Elevated levels of GSH were also associated with resistance to platinum-based drugs, including CDDP [55]. Godwin et al. [56] reported a correlation between resistance of CDDP in human ovarian cancer and the increase of GSH cellular level, as a marker of CDDP-induced resistance. The mechanisms involved in CDDP resistance are intensively studied but not fully elucidated. There are hypothesis that sustain the role of GSH in conjugation reaction of CDDP prior to their efflux through multidrug resistance proteins (MRPs) transporters in mammalian cells but also its function as copper chelator or redox-regulating cytoprotector, properties with implications on CDDP resistance process [57].

Besides the antioxidant defence offered by GSH, cells are protected by the enzyme glutathione-S-transferase (GST) which participates in cell detoxification by catalysing the conjugation of GSH with electrophilic compounds (endogenous and exogenous) to form glutathione-S-conjugates [58,59].

**Figure 11:**
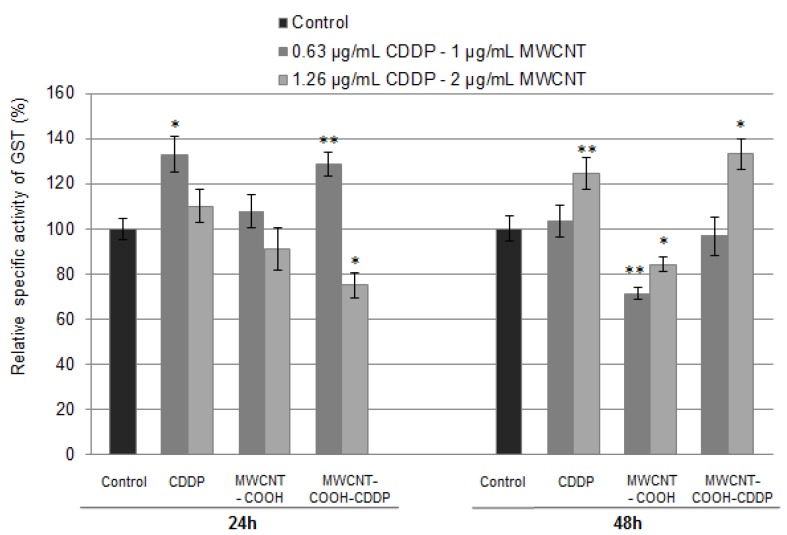
Relative specific activity of GST after MDA-MB-231 cells exposure to MWCNTs (1, 2 µg/mL) and CDDP (0.63, 1.26 µg/mL) for 24 h and 48 h. The results are calculated as the mean ± SD of 3 replicates and represented relative to control. * *p* < 0.05, ** *p* < 0.01 vs. control.

The specific activity of GST was assessed in breast cancer cells after exposure for 24 h and 48 h to 1, 2 µg/mL MWCNTs and 0.63, 1.26 µg/mL CDDP. As can it be observed from Figure 11, after 24 h, GST activity significantly increased in cells treated with a concentration of 1 µg/mL MWCNT-COOH-CDDP and 0.63 µg/mL CDDP respectively and decreased in cells exposed to 2 µg/mL MWCNT-COOH-CDDP, respectively 1.26 µg/mL CDDP. No obvious changes of GST activity were observed after 24 h of exposure to MWCNT-COOH but a significant decrease was induced after 48 h. Interestingly, after 48 h a switch in GST activity was observed. Thus, in cells treated with 1 µg/mL MWCNT-COOH-CDDP and 0.63 µg/mL free CDDP, the GST activity dropped reaching the level registered in control cells and it rises in cells treated with 2 µg/mL MWCNT-COOH-CDDP and 1.26 µg/mL free CDDP. These variations possible suggest the development of drug resistance against treatment with CDDP. High levels of GST were already associated with resistance to CDDP [59]. Also, it was demonstrated that GST-mediated drug resistance can be blocked using GST inhibitors which enhance CDDP efficiency in lung cancer treatment [60]. Inhibition of GST activity and GSH depletion after 24 h accompanied by significant increases of these parameters after 48 h suggest that cancer cells managed to counteract the oxidative stress and kept the steady state evading the activation of cancer cell death.

In cells, the expression of GST is regulated by a stress-responsive transcription factor called the nuclear factor erythroid 2-related factor 2 (Nrf2). The activation of Nrf2 factor is often linked to perturbation of cellular thiol status and/or oxidative stress including intracellular glutathione depletion or ROS increases [61]. In normal conditions, Nrf2 is located in cytoplasm and it is conjugated with a homodimer protein Kelch-like ECH-associated protein 1 (Keap1). This interaction promotes ubiquitination of Nrf2 protein which is then directed to proteasomal degradation. In oxidative stress conditions, ubiquitination is inhibited, Keap1 protein become saturated with Nrf2 and, consequently, Nrf2 protein is translocated in nucleus where it triggers cellular defence mechanisms by its binding to antioxidant response elements (ARE) [62,63].

**Figure 12:**
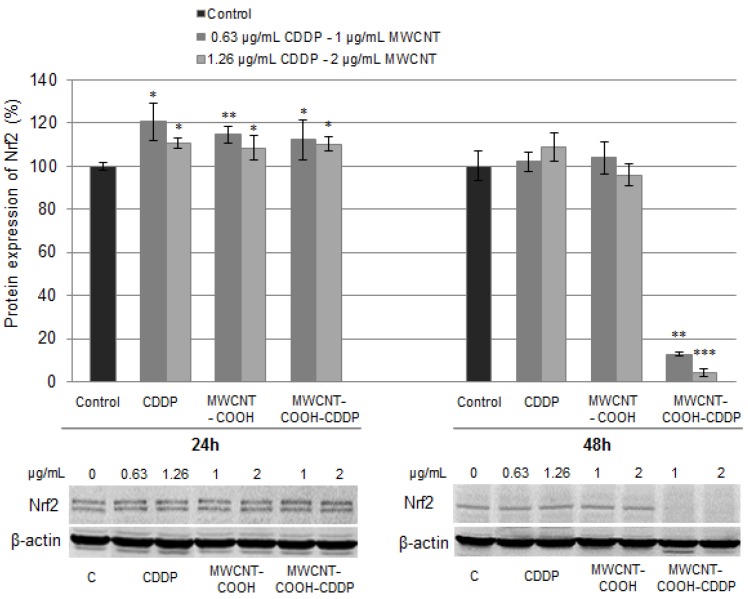
Relative protein expression of Nrf2 after the exposure of MDA-MB-231 cells to MWCNTs (0.5–4 µg/mL) and CDDP (0.316–2.52 µg/mL) for 24 h and 48 h. The graph is the correspondent quantification of blots images presented below. The results are calculated as the mean ± SD of 3 replicates and represented relative to control. * *p* < 0.05, ** *p* < 0.01, *** *p* < 0.001 vs. control.

Following the treatment, we quantified the expression of poly-ubiquitinated Nrf2 (~100 kDa) in MDA-MB-231 cells in accordance with Lau et al. [64]. The Nrf2 polyclonal antibody (H-300) recognizes only two of the Nrf2 isoforms [65] which appear as two closed bands on blot membrane. Thus, after blotting we detected a significant increase of Nrf2 expression after 24 h exposure in all treated cells (Figure 12). Surprisingly, after 48 h, only one of the Nrf2 isoforms could be seen on the membrane. Moreover, a significant down-regulation of poly-ubiquitinated Nrf2 expression was noticed in cells exposed to MWCNT-COOH-CDDP suggesting the activation of Nrf2 by its translocation to the nucleus [66]. In normal cells, a down-regulation of poly-ubiquitinated Nrf2 protein was also observed after the treatment with 1, 2 µg/mL MWCNT-COOH-CDDP and 1.26 µg/mL CDDP for 48 h, with a more pronounced decrease for MWCNTs loaded with CDDP sample (Figure S4). Considering the mechanism of activation, the decreased expression of poly-ubiquitinated Nrf2 protein can be associated with an anti-oxidative defence mechanism of normal cells in the presence of high levels of produced ROS.

This in correlation with the decrease of ROS generation and increase of GSH level, indicates a mechanism of cellular defence against oxidative stress in MDA-MB-231 breast cancer cells which lead to drug resistance. Not the same expression of Nrf2 was observed in cells exposed to free CDDP. A possible explanation might be that a smaller amount of CDDP passed the membrane and was metabolized in breast cancer cells compared with the CDDP transported in MWCNTs.

On the other hand, several studies reported that various drugs including CDDP induce resistance in cancer cells through Nrf2 activation [66,67]. Bao et al. [68] reported the resistance of ovarian carcinoma cells under CDDP exposure through activation of autophagy induced by Nrf2 and after they showed that knockdown of Nrf2 can sensitive CDDP-resistant cancer cells to CDDP treatment and induce apoptosis. Thus, activation of Nrf2 after 48 h exposure to MWCNT-COOH-CDDP could suggest the induction of cellular resistance.

### 3.9. Cell Death Inhibition

**Figure 13:**
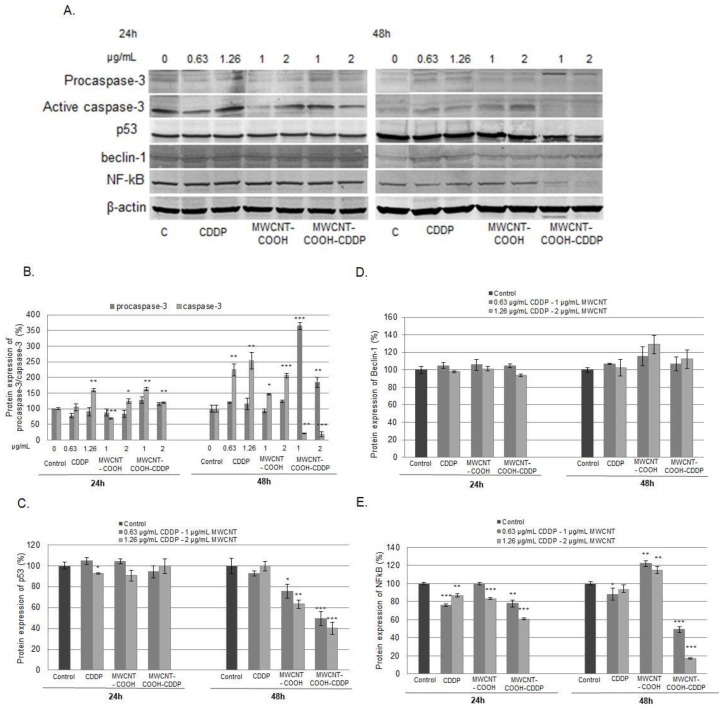
Relative protein expressions of procaspase-3/caspase-3 (**B**), p53 (**C**), Beclin-1 (**D**) and NF-κB (**E**) after the exposure of MDA-MB-231 cancer cells to 0.63, 1.26 µg/mL CDDP and 1, 2 µg/mL MWCNT for 24 h and 48 h. The graphs are the correspondent quantification of blot images (**A**). The results are calculated as the mean ± SD of 3 replicates and represented relative to control. * *p* < 0.05, ** *p* < 0.01, *** *p* < 0.001 vs. control.

Markers of apoptotic cell death (caspase-3, p53) were analysed after breast cancer cells exposure to 0.63, 1.26 µg/mL CDDP and 1, 2 µg/mL MWCNT for 24 and 48 h, to better understand and clarify the outcomes of our experiment (Figure 13B,C). Moreover, Beclin-1 and NF-κB protein expression were evaluated as alternative pathways leading to cell death (Figure 13D,E).

After 24 h of exposure, the expression of active caspase-3 increased after the treatment with 1.26 µg/mL CDDP and 2 µg/mL MWCNT-COOH and with both tested concentrations of MWCNT-COOH-CDDP. But after 48 h, the expression of caspase-3 was significantly increased when the cells were exposed to CDDP and MWCNT-COOH. Interesting, when the cells were treated with the MWCNT-COOH-CDDP sample, an inhibition of active caspase-3 associated with an increase of procaspase-3 expression was recorded (Figure 13B). Considering this, the results suggest that individual components, CDDP and MWCNT-COOH, could lead to apoptosis in MDA-MB-231 breast cancer cells but not their combination. Cell death induced by CDDP was reported also in other papers, apoptosis being described as the main mechanism of action of this chemotherapeutic [18,20].

Given its ability to induce both cell-cycle arrest and cell death, the expression of transcription factor p53 was also a parameter of interest for our study. P53 activation induces the G1 arrest by up-regulation of p21WAF1/CIP1/Sdi1, an inhibitor of the cyclin dependent kinases (CDKs) and also G2/M arrest and down regulation of cyclin A expression, causing a break on cell cycle progression through the S phase. Among other functions, p53 protein can promote apoptosis process through its activation and transcription of genes that contain p53-specific elements. These genes encode proteins associated to cellular membrane, cytosol or mitochondria (Bax) [69]. Apoptosis induced by p53 pathway involves also caspases activation thus leading to release to cytochrome c and formation of apoptosome, a complex protein specific to apoptosis [70].

Our results showed no significant modifications of p53 expression after 24 h of treatment exposure. After 48 h, a pronounced down-regulation of p53 was noticed in cells treated with MWCNT-COOH-CDDP compared with the other samples which confirms the cell death inhibition in breast cancer cells (Figure 13C).

The inhibition of cell death mechanisms was underlined also by the unaltered expression of Beclin-1 protein after MDA-MB-231 cells treatment with CDDP, MWCNT-COOH and MWCNT-COOH-CDDP (Figure 13D). Beclin-1 is a protein with a central role in autophagy, as it intervenes in every major step of autophagic pathways, from autophagosome formation, to autophagosome/endosome maturation [71]. Autophagy can be an alternative pathway for cancer cells to survive with altered metabolism and in the hostile tumour microenvironment [72]. Our results demonstrated that MDA-MB-231 cells did not undergo autophagy after exposure neither for individual components, either for their combination.

The expression of NF-κB protein (p65 subunit) was measured in MDA-MB-231 cells to estimate DNA damage. After 24 h of exposure, the expression of NF-κB decreased in cells treated with all tested suspensions, except for the lowest dose of MWCNTs were the level was unchanged. However, we noticed a more pronounced NF-κB down-regulation in cells treated with MWCNT-COOH-CDDP (2 µg/mL MWCNTs/1.26 µg/mL CDDP). Starting with 48 h, the expression of NF-κB encountered a significant down-regulation when the cells were exposed to MWCNT-COOH-CDDP sample. Moreover, an interesting recovery of NF-κB expression was observed after the treatment with CDDP and a significant increase when cells were exposed to MWCNT-COOH (Figure 13E).

NF-κB is a transcription factor activated through the cooperation of ataxia telangiectasia mutated (ATM) and NF-κB essential modulator (NEMO) in response to DNA lesions. When activated NF-κB is translocated from cytoplasm to the nucleus where regulates the expression of pro- and anti-apoptotic factors and ultimately protecting the cell from apoptosis [73,74]. Interestingly, DNA intercalators, which alter the structure of DNA, also induce activation of NF-κB, leading to a significant increase in DNA damage and ultimately, apoptosis [73]. Considering this, the down-regulation of NF-κB protein expression in MDA-MB-231 cells exposed to MWCNT-COOH-CDDP system is far from indicating DNA damage. Rather, the inhibition of NF-κB expression have suggested us that this could be a pathway of cancer cells to develop a DNA-binding independent resistance. Several researchers attempted to examine the association between the status of NF-κB expression and resistance to chemotherapy in several tumours. A growing number of evidence indicated that activation of NF-κB is associated with resistance to apoptosis. Regarding to the association between NF-κB activation/expression and chemoresistance, Antoon et al. reported that the breast cancer chemo-resistance cell line MCF-7TN-R overexpressed NF-κB [75]. Also, a strong nuclear localization of NF-κB was observed after the development of acquired platinum-resistance in bladder cancer which led to belief that regulation of the NF-κB pathway might be a potent therapeutic target in platinum-resistant bladder cancer [76].

By studying the biological effects of MWCNT-COOH-CDDP we found that this combination induces resistance in TNBC cells to high ROS levels, escaping cell death. Free CDDP has been also tested in TNBC by other researchers and the disadvantage of resistance development was also noticed. Many studies tried to identify the resistance mechanisms in CDDP-resistant TNBC cell lines. For example, Pendleton, C.S. from Vanderbilt University, in his thesis entitled “Mechanisms of Cisplatin Resistance in Triple Negative Breast Cancer,” 2014 [77] found that resistance mechanisms involve some aspect of attenuation of apoptosis after CDDP treatment and provided strong preliminary evidence for the continued study of caspase-14 and the MEK/ERK signalling axis as mechanisms of CDDP resistance in TNBC. Another research group, showed that activation of EGFR and IGF1R and their downstream kinase Akt signalling pathway was associated with resistance induced by long-term treatment with CDDP in the TNBC cell line HCC38 and in MDA-MB-231 [78]. Others, found that intrinsic resistance to CDDP is associated with P-Glycoprotein down-regulation in NCI-H-446 cells [79].

We studied the effects of MWCNT-COOH-CDDP in parallel with of individual components on MDA-MB-231 cells. Comparing the results, we found that toxicity of MWCNT-COOH-CDDP was lower related to the one of free CDDP in breast cancer cells. In order to understand the mechanism activated in cellular resistance to MWCNT-COOH-CDDP we further analysed other key proteins to discover their implication in drug resistance. One of the targeted proteins was Nrf2. In this regard, we found also that MWCNT-COOH-CDDP induced activation of Nrf2. In addition, we discovered changes in the expression of several other proteins in breast cancer cells treated with MWCNT-COOH-CDDP such as: down-regulation of p53, up-regulation of pro-caspase 3, inhibition of active caspase 3 and down-regulation of NF-κB. Also we showed that expression of the Beclin1 protein which is a marker of autophagy, was not changed in our case.

The effects of MWCNT-COOH-CDDP were tested also on normal cells. By analysing the release of LDH in culture medium of cells exposed to MWCNT-COOH-CDDP, we found an increase of LDH leakage after 48 h only in cancer cells. In normal cells, damage of cellular membrane was recorded only for free CDDP which might suggest that combination with MWCNTs was unable to disrupt the membrane of these cells. However, the ROS production induced by MWCNT-COOH-CDDP was higher in normal cells compared to cancer ones. Moreover, Nrf2 protein was activated in both types of cells but, interestingly, in normal cells was also activated in response to a high dose of free CDDP, as a protective mechanism. Considering the results, is difficult to make affirmations regarding the selectivity to cancer cells to MWCNT-COOH-CDDP. However, for normal cells was proved to be resistant to MWCNT-COOH-CDDP treatment.

It is known that cytotoxic chemotherapy agents exert their effects by disrupting the cell cycle by one or more processes. As cancer cells undergo rapid cell divisions, they are generally more susceptible to drugs than normal cells. Unfortunately, cancer cells develop insensibility to growth inhibitory signals and evade cell death. In the same time, the drugs could also interfere with normal cell divisions [80].

In this regard, the resistance of cancer cells can be exploited to kill resistant cells selectively, while sparing sensitive normal cells. By now, several studies aimed to improve the CDDP treatment by increasing sensitivity of cancer relative to normal cells. Gorsic et al. [81] used an inhibitor of *EPS8*, such as mithramycin A to improve the CDDP sensitivity to lung cancer cells compared to normal cells. Recently, Huang et al. [82] showed that FOLR1 which is highly expressed in ovarian cancer but reduced following drug resistance could be used to increases sensitivity to CDDP treatment in ovarian cancer cells.

Here, we show that transport of CDDP in MWCNTs does not overcome the TNBC cells resistance but could act by modulation of different signalling pathways compared with free CDDP. Overall, the MWCNT-COOH demonstrated a good biocompatibility and high efficiency for CDDP transportation across the membrane but we still need to conduct research investigations to properly address the problem of drug resistance. These results, could represent an important step in understanding and development of new strategies for finding targets to reverse, overcome or prevent the drug resistance in cancer cells.

## 4. Conclusions

In this study, we referred to platinum-based drug that are used in cancer therapy (CDDP) that have been encapsulated in carboxyl functionalized MWCNTs. After chemical functionalization of MWCNT-COOH groups were highlighted by FTIR measurements, the procedure leading to carbon nanotubes cover by chemotherapeutic agent. EDX analysis indicated the presence of Pt ions on the surface of MWCNTs. The ICP-MS analysis indicated the presence of total amount of Pt (encapsulated and trapped on the surface of carbon nanotubes). CDDP release occurs rapidly in the first few hours of immersion in PBS not exceeding its pH.

The in vitro analyses on MDA-MB-231 cell line led to the following conclusions regarding the MWCNT-COOH-CDDP: (i) First, a dose above 4 µg/mL MWCNTs and 2.52 µg/mL CDDP of CDDP-loaded MWCNT-COOH is able to induce reduction of cell viability, morphological changes and loss of membrane integrity starting with 24 h within breast cancer cells; (ii) Second, ROS are produced in MDA-MB-231 cells after 24 h of exposure even at lower doses but their level decreases over time as result of cancer cell protection mechanisms; (iii) Third, the antioxidant defence was activated in breast cancer cells after 48 h through the increase in GSH pool and GST activity (iv) Fourth, MWCNT-COOH-CDDP leads to activation of Nrf2 in breast cancer cells after 48 h as a mechanism for triggering drug resistance; (v) Lastly, the inhibition of apoptosis and absence of DNA damage were promoted by suppression of caspase-3, p53 and NF-κB expression in breast cancer cells treated with MWCNTs loaded with CDDP.

Our study showed that combination of MWCNTs with CDDP activates different pathways in TNBC cells compared to individual components, information which might contribute to the understanding of TNBC cells resistance and the guidance of further therapeutic strategies approaches.

## Supplementary Materials

The following are available online at http://www.mdpi.com/1999-4923/10/4/228/s1, Figure S1: The level of LDH released in culture medium by non-tumour MRC-5 cells after 24 h and 48 h of exposure of various concentrations of CDDP (0.316–2.52 µg/mL) and MWCNT (0.5–4 µg/mL), Figure S2: Bright-field images presenting MRC-5 cells morphology after exposure to various concentrations of CDDP (0.316–2.52 µg/mL) and MWCNTs (0.5–4 µg/mL) for 24 h and 48 h, Figure S3: Relative level of ROS generated after the exposure of MRC-5 cells to CDDP (0.316–2.52 µg/mL) and MWCNTs (0.5–4 µg/mL) for 24 h and 48 h, Figure S4: Relative protein expression of Nrf2 after non-tumor MRC-5 cells exposure to 0.63, 1.26 µg/mL CDDP and 1, 2 µg/mL MWCNTs for 48 h.
