# Supplementary material for: Cisplatin Loaded Multiwalled Carbon Nanotubes Induce Resistance in Triple Negative Breast Cancer Cells
Source: Pharmaceutics. 2018 Nov 13;10(4):228. doi: 10.3390/pharmaceutics10040228 (PMC6321179; doi:10.3390/pharmaceutics10040228)
Supplement: Supplementary file 1 [file pharmaceutics-10-00228-s001.pdf]

pharmaceutics

MDPI

Supplementary Materials: Cisplatin Loaded Multiwalled Carbon Nanotubes Induce Resistance in Triple Negative Breast Cancer Cells

Madalina Andreea Badea, Mariana Prodana, Anca Dinischiotu, Carmen Crihana, Daniela Ionita * and Mihaela Balas

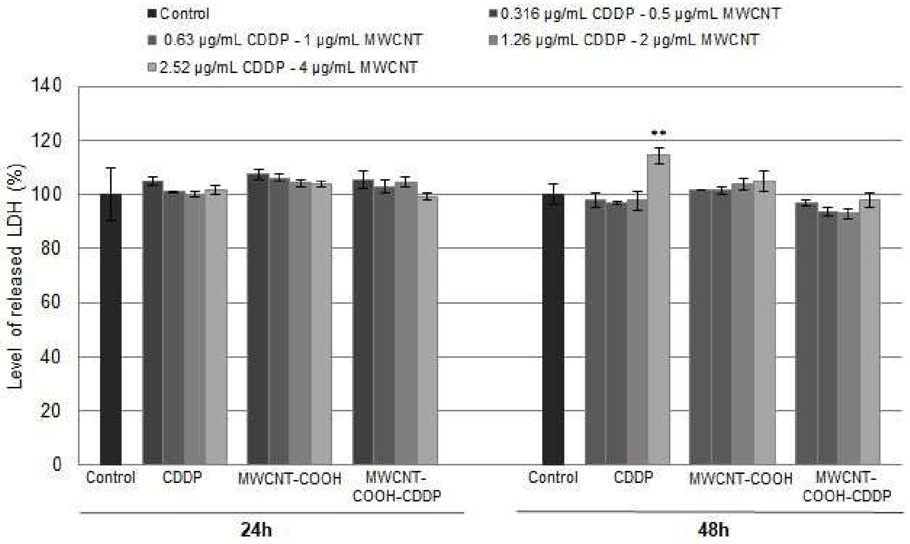

Figure S1. The level of LDH released in culture medium by non-tumor MRC-5 cells after 24 h and 48 h of exposure of various concentrations of CDDP (0.316 – 2.52 μg/mL) and MWCNT (0.5 – 4 μg/mL). The results are calculated as the mean ± SD of 3 replicates and represented relative to control (untreated cells). ** $p < 0.01$ vs. control.

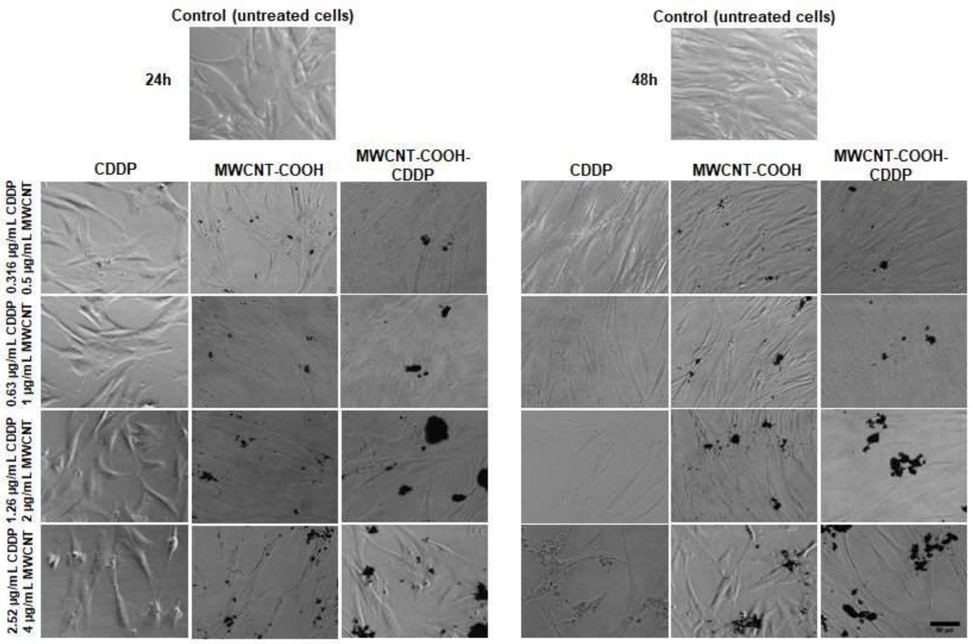

Figure S2. Bright-field images presenting MRC-5 cells morphology after exposure to various concentrations of CDDP (0.316 – 2.52 μg/mL) and MWCNTs (0.5 – 4 μg/mL) for 24 h and 48 h. Control represents untreated cells. Aggregates of MWCNTs are visible over the cell fields. Scale bar: 50 μm.

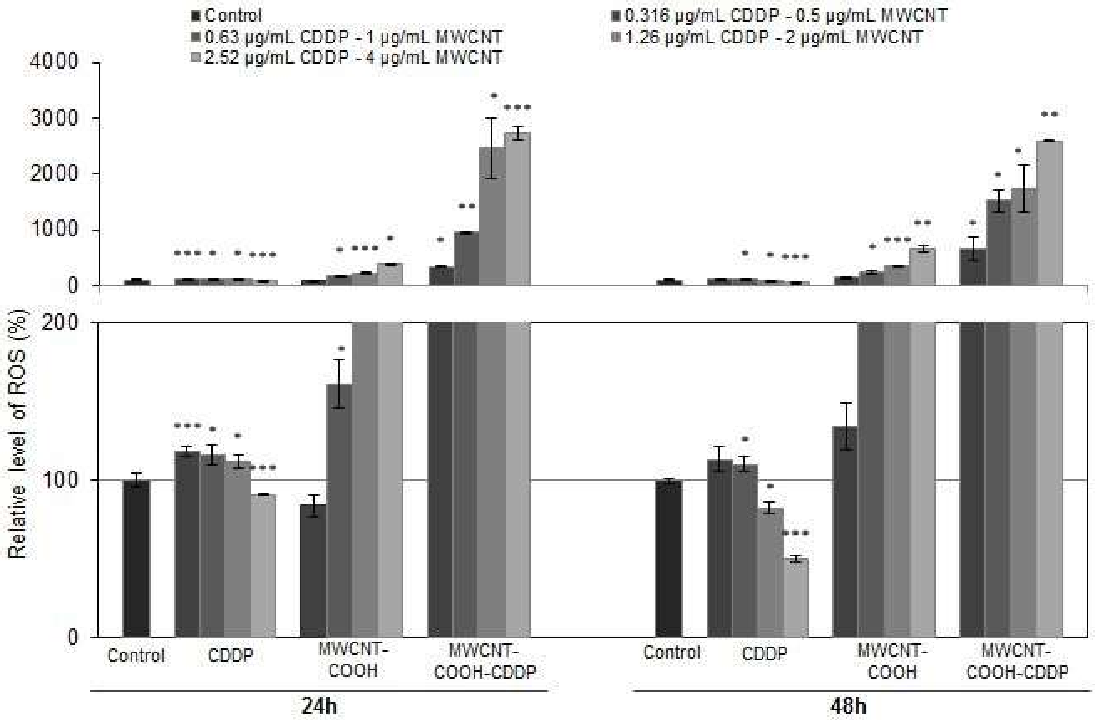

Figure S3. Relative level of ROS generated after the exposure of MRC-5 cells to CDDP (0.316 – 2.52 µg/mL) and MWCNTs (0.5 – 4 µg/mL) for 24 h and 48 h. The results are calculated as the mean ± SD of 3 replicates and represented relative to control. * $p < 0.05$, ** $p < 0.01$, *** $p < 0.001$ vs. control. The lower graph presents a magnified image of the scale range between 0 - 200 from the upper graph.

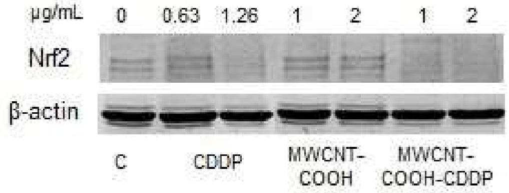

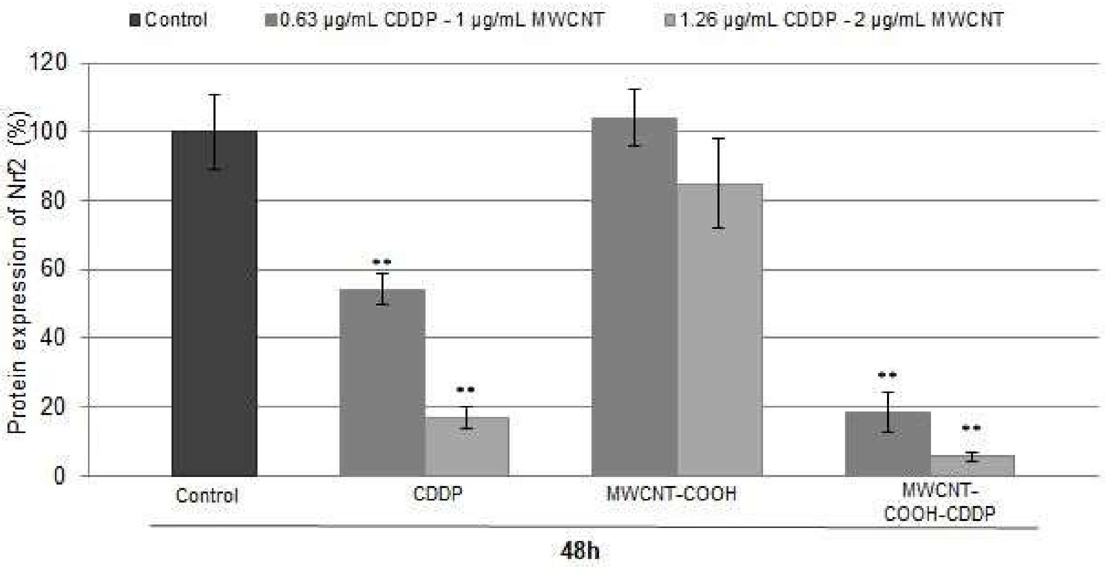

Figure S4. Relative protein expression of Nrf2 after non-tumor MRC-5 cells exposure to 0.63, 1.26 µg/mL CDDP and 1, 2 µg/mL MWCNTs for 48 h. The graph is the correspondent quantification of blots images. The results are calculated as the mean ± SD of 3 replicates and represented relative to control. ** $p < 0.01$ vs. control.
